# The development of amivantamab for the treatment of non-small cell lung cancer

**DOI:** 10.1186/s12931-023-02558-4

**Published:** 2023-10-25

**Authors:** Danielle Brazel, Misako Nagasaka

**Affiliations:** 1 Scripps Clinic/Scripps Green Hospital, La Jolla, USA; 2 University of California Irvine Department of Medicine, Orange, CA USA; 3 Chao Family Comprehensive Cancer Center, Orange, CA USA; 4 St. Marianna University School of Medicine, Kawasaki, Japan

**Keywords:** Amivantamab, Epidermal growth factor receptor (*EGFR*), Mesenchymal-epithelial transition factor (*MET*), Non-small cell Lung cancer, Tyrosine kinase inhibitors

## Abstract

Non-small cell lung cancer (NSCLC) patients with sensitizing oncogenic driver mutations benefit from targeted therapies. Tyrosine kinase inhibitors are highly effective against classic sensitizing epidermal growth factor receptor (*EGFR*) mutations, such as exon 19 deletions and exon 21 L858R point mutations. Conversely, *EGFR* exon 20 insertions (exon20ins) are resistant to the traditional *EGFR* tyrosine kinase inhibitors (TKIs). In May 2021, the US Federal Drug Administration (FDA) provided accelerated approval to amivantamab (Rybrevant) in adults with locally advanced or metastatic NSCLC with *EGFR* exon20ins after treatment with platinum-based chemotherapy. Amivantamab was the first *EGFR/MET* bispecific antibody to be approved specifically for *EGFR* exon20ins where there was an unmet need. Furthermore, amivantamab is being evaluated in additional settings such as post osimertinib in sensitizing *EGFR* mutations as well as in *MET* altered NSCLC. Here we discuss amivantamab in regard to its mechanism of action, preclinical and clinical data, and clinical impact for patients with *EGFR* exon20ins NSCLC and beyond.

## Introduction

Non-small cell lung cancer (NSCLC) is the leading cause of cancer-related mortality worldwide [1, 2]. Increased access to DNA sequencing for targetable mutations and rapid advancements in targeted therapeutic options have improved outcomes in many subtypes of NSCLC [3–5]. Drugs targeting molecular oncogenic drivers have improved efficacy and tolerability of treatment for NSCLC patients. *EGFR* is a transmembrane cell surface receptor with downstream effects that regulate cell proliferation and apoptosis [6]. In normal cells, *EGFR* signaling is responsible for DNA synthesis and cellular proliferation, however, surplus activity results in uncontrolled cellular growth and tumorigenesis [7]. *EGFR* mutations generally favor the active state leading to pro-survival and antiapoptotic signals, even without the presence of a ligand [8, 9]. *EGFR* is an attractive target for therapeutic development as *EGFR*-mutated tumors become dependent on the *EGFR* pathway and its downstream effects for survival [6]. *EGFR*-mutated NSCLC is found on sequencing of 20% of Caucasians, up to 50% of Asian patients [4, 10]. and globally, *EGFR* mutations account for 23–30% of NSCLC activating mutations [11, 12].

Classic *EGFR* mutations (exon 19 deletions or exon 21 L858R substitutions) represent 85% of *EGFR* mutations [13]. *EGFR* exon20ins (exon20ins) consist of either point mutations or insertions of 3–21 base pairs [14]. and are present in 4-to-12% of *EGFR*-mutated NSCLC [15]. NSCLC driven by *EGFR* exon20ins portends a worse prognosis and shorter overall survival than classic sensitizing *EGFR* mutations such as exon 19 deletions and exon 21 L858R point mutations [16, 17]. Because of its structure, the active conformation with the C-helix in an inward position, forming a rigid and inflexible structure that locks the *EGFR* molecules in active conformation without ligand binding [12 Yasuda], *EGFR* exon20ins are classically resistant to first-, second-, and third-generation *EGFR* tyrosine kinase inhibitors (TKIs) and prior to approval of amivantamab in 2021, there were no Federal Drug Administration (FDA) approved targeted therapeutic options. In May 2021, the FDA granted accelerated approval to amivantamab (Rybrevant) in adult NSCLC patients with locally advanced or metastatic *EGFR* exon20ins-positive disease following platinum-based chemotherapy.

Mesenchymal-epithelial transition (*MET*) is a tyrosine kinase receptor for the ligand hepatocyte growth factor (HGR) and is frequently expressed by epithelial cells of solid organs. Dysregulation of the *MET* pathway results in proliferation, survival, invasion, and metastasis of tumor cells. *MET* activation is both a primary oncogenic driver mutation and could be a secondary mechanism of drug resistance, making the *MET* pathway an attractive therapeutic target [18, 19]. *MET* aberrations can occur as overexpression, amplification or mutations. *MET* is overexpressed in 20%, [20] amplified in 1–5%, [21] and exon 14 skipping mutations (*MET*ex14) occur in 3–4% of NSCLC tumors [22–24]. *MET* rearrangements have been detected in several cancer types including NSCLC and glioblastomas [25, 26]. In chromosomal translocations, the fusion typically includes a dimerization domain resulting in constitutive activation. Although the TPR-MET fusion was first identified, [27] a *ST7-MET* fusion was reported as an acquired resistance mechansim to the third-generation TKI lorlatinib in a NSCLC patient with dual *ALK-MET* aberrations [28]. Within NSCLC tumors, *MET*ex14 skipping mutations are most frequent in sarcomatoid carcinoma (4.9–31%), adenosquamous carcinoma (5%), adenocarcinoma (3%), and squamous cell carcinoma (2%) [29–33] and are more common in patients over 70 years old, women, and never-smokers. *MET* aberrations are associated with poor prognosis [34–36]. *MET* exon 14 skipping mutations occur at high allele frequency and can co-occur with *TP53*, *MDM2*, *CDK4*, and *HMGA2* co-amplifications while *MET*-amplified patients have co-occurring *NRAS* and *KRAS* mutations [37]. Conversely, one study of 30 patients with *MET*ex14 aberrations found no overlap with mutations in *KRAS, EGFR, ERBB2, ALK, ROS1, or RET* [29]. *MET*ex14 skipping mutations are associated with worse overall survival [38].

*MET* amplification has been shown to bypass *EGFR* signaling pathways and confer resistance to osimertinib [39, 40]. *MET* amplification was found in 15% of samples at disease progression on osimertinib [41]. *MET* amplification has been described in cases of rapid and prolonged response to crizotinib [42]. *MET*-mutated tumors are also associated with a worse prognosis [43]. Additional *MET* aberrations included impaired *MET* receptor degradation, *MET* fusion, and *MET* overexpression.

Upregulation of the *EGFR* signaling pathway has been shown as a mechanism of resistance to *MET* TKIs [44, 45]. *MET* amplification is associated with resistance in 50–60% of first- and second-generation *EGFR* TKIs [46–48] and 15–19% of third-generation *EGFR* TKIs [41, 49]. *EGFR* and *MET* are co-expressed in 70% of *EGFR* mutations [50, 51]. In contrast, normal cells almost never concomitantly express both receptors [52, 53]. Interactions between *EGFR* and *MET* signaling pathways is well documented in the literature and are involved in both oncogenic signaling as well as tumor microenvironment remodeling [54–56]. Both *EGFR* and *MET* signal through the same pathways, possibly explaining frequent resistance upon inhibition of only one of these receptors [57]. The interplay between these pathways suggests that simultaneously inhibiting both oncogenes may reduce resistance to *MET*- or *EGFR*-targeted agents.

The first *MET* inhibitor, crizotinib, was approved in 2011 for *ALK*-rearranged NSCLC and since that time, *MET*-targeted drugs including capmatinib and tepotinib have been approved for NSCLC harboring *MET* exon 14 skipping mutations. Capmatinib is a type 1b *MET* inhibitor with a mechanism similar to that of crizotinib. The phase II GEOMETRY study, reported an objective response rate (ORR) of 41% (95% CI 29–53) in pretreated patients with *MET* exon 14 skipping mutations and 68% (95% CI 48–84) in treatment naïve patients [58]. The duration of response (DOR) was 9.7 months and 12.6 months, respectively. Among patients with *MET* amplification and a gene copy number of 10 or higher, OR was seen in 29% (95% CI, 19 to 41) of pretreated patients and in 40% (95% CI, 16 to 68) of treatment naïve patients. [51]. Tepotinib is a TKI that selectively binds *MET* to promote tumor cell death and in the phase II VISION trial, tepotinib had an ORR 46% (95% CI 36–57) with median DOR 11.1 months (95% CI 7.2-NR) [59].

In this review, we discuss the unique structure, pharmacodynamics, and pharmacokinetics of amivantamab as well as its indication towards *EGFR* exon20ins and beyond, focusing on dual inhibition of *EGFR* and *MET*, which could be employed for the treatment of *MET*-altered tumors as well as those with sensitizing *EGFR* mutations who have progressed on *EGFR* TKIs.

## Structural characteristics and mechanism of action of amivantamab

Amivantamab (JNJ-61,186,372, Rybrevant, Janssen Biotech, Inc) is a fully human Fc-active immunoglobulin G1 (IgG1) bispecific antibody against both epidermal growth factor (*EGF*) and *MET* receptors. Amivantamab consists of two arms; one binds the extracellular domain of *EGFR* to block binding between the receptor and its ligand EGF while the other arm blocks *HGF* ligand from binding to the *MET* receptor. Amivantamab induces degradation of both receptors in vivo, broadening its mechanism of action to include ligand-independent driven disease [15, 60]. This results in stopping downstream signaling of pro-growth and pro-survival proteins. Amivantamab simultaneously inhibit *EGFR* as well as one of the more common mechanisms of resistance to *EGFR* targeting therapy through the *MET* pathway. This combined inhibition has the potential to enhance depth and duration of response for patients with these mutations.

In addition to the direct inhibitory effects as a bispecific antibody, amivantamab also appears to work with the human immune system. Indeed, amivantamab has a low fructose backbone to enhance binding to FcYRIIIa/CD16a [61]. The FcYRIIIa/CD16a receptor on NK cells, monocytes, and macrophages triggers antibody-dependent cell-mediated cytotoxicity (ADCC) of NSCLC cells. This unique structural design permits amivantamab to eliminate antigen-expressing tumor cells through ADCC, induce trogocytosis as well as antibody dependent cellular phagocytosis and antibody dependent cytokine release. This activity results in receptor-antibody complex endocytosis and removal via lysosomal trafficking [61].

## Pharmacodynamic properties

Amivantamab has been shown to bind the extracellular domains of *EGFR* and *MET* receptors with binding affinity (K_D_) of 1.43 and 0.04 nM, respectively in preclinical studies. Amivantamab binds human *EGFR* and *MET* with EC50 values of 0.38 nM 0.27 nM, respectively. Amivantamab was selected from a panel of bispecific anti-*EGFR* and anti-*MET* molecules. Bispecifics with higher affinity to *MET* were favored to reduce binding to cells with normal *EGFR* expression. The affinity for *MET* was seen with equilibrium dissociation constant [Kd] of 40pmol/L. It is proposed that the high affinity for *MET* plus low affinity for *EGFR* helps overcome resistance while also decreasing wild-type *EGFR*-associated toxicity.

In vitro studies also found at doses ≥ 700 mg, complete and durable saturation of both *EGFR* and *MET* receptors occurred [62].

## Pharmacokinetics

Data from in vivo trials showed at doses between 350 and 1750 mg, amivantamab exposure increased proportionally. Steady state was achieved by the ninth infusion. Amivantamab concentration increased rapidly during cycle 1 with steady state observed by cycle 4 [62]. The half-life of amivantamab was 11.3 (± 4.53) days with a median volume of distribution of 5.13 L.

Dosing recommendations are based on target saturation that were calculated using baseline body weight. For patients with baseline body weight < 80 kg, the recommended dose of amivantamab is 1050 mg. For patients above 80 kg at baseline, the recommended dose is 1400 mg. Amivantamab is administered intravenously weekly for the first five weeks then every two weeks until disease progression or toxicity. There was no clinically meaningful difference in amavantamab exposure across age, gender, race, creatinine clearance, or hepatic impairment.

## Preclinical studies

Preclinical studies have confirmed the unique characteristics of amivantamab. The low fructose backbone of amivantamab appears to enhance ADCC through stronger binding to the F-c domain [63]. Indeed, studies in mice confirmed that tumors treated with amivantamab had lower *EGFR* and *MET* receptor expression as a result of receptor internalization and trogocytosis [60].

Preclinical studies of Ba/F3 cell lines containing *EGFR* exon20ins have found that amivantamab decreased EGF and *MET* receptor expression [15]. All five exon20ins studied (V769_D770insASV, D770delinsGY, H773_V774insH, Y764_V765insHH, and D770_N771ins- SVD) demonstrated a dose-dependent decrease in viability. The proposed mechanism of action is inhibition of cell proliferation through decreased pERK, pAkt, and p-S6 [15]. Additionally, amivantamab induced apoptosis via upregulating proapoptotic proteins including *BCL2*-interacting mediator of cell death (BIM) and cleaved caspase-3.

HCC827 is a lung adenocarcinoma cell line with an acquired E746_A750 deletion in the *EGFR* tyrosine kinase exon 19 domain. Data from HCC827 cell lines have found superior antitumor activity of amivantamab compared to TKI erlotinib and the *MET* inhibitor crizotinib. By day 34, tumor growth was inhibited 99.8% (p < 0.05) with a durable response 8 weeks after amivantamab discontinuation [64]. In experiments with xenograft models amivantamab was more efficacious than either cetuximab or poziotinib [15].

Additionally, amivantamab has preclinical data from resistant cell lines. Within cell lines of *EGFR* activating mutations (i.e. L858R), with *EGFR* resistance mutations (i.e. T790M)or *MET* amplification, amivantamab blocked ligand from binding its receptor [65]. This showed the antitumor activity of amivantamab even in tumors with mechanisms of resistance to *EGFR* targeted therapy as well as the historically difficult to target *MET* amplification. Further, amivantamab results in decreased cell surface *EGFR* and *MET* receptors both in vitro and in vivo [60]. Importantly, amivantamab remains effective when bound to either *EGFR* or *MET* receptors alone [60, 63].

## Clinical trials

### EGFR exon20ins

#### Phase I

Amivantamab was granted accelerated FDA approval to those with NSCLC patients with tumors harboring *EGFR* exon20ins post platinum based therapy on May 21, 2021. This was based on CHRYSALIS, a phase I, multicenter, open label, dose-escalation clinical trial (NCT02609776) [62]. Patients received amivantamab IV weekly for five weeks then every two weeks until disease progression or untolerable toxicity. The primary outcome was overall response rate (ORR) with secondary outcomes of interest including clinical benefit rate, duration of response (DOR), progression-free survival (PFS), and overall survival (OS). The trial overall enrolled 362 patients with median age of 62 years, 48% women, and 49% Asian. All study participants were platinum-pretreated with a median of 2 (range 1–7) previous therapies. During the dose escalation portion, no maximum tolerated dose was identified up to the highest studied dose of 1750 mg. Weight-based dosing was selected at a dose of 1050 mg for patients under 80 kg or 1400 mg for patients 80 kg or more due to the safety, pharmacokinetic, and pharmacodynamic properties. In those with NSCLC harboring *EGFR* exon20ins post platinum-based therapy (N = 114), the ORR was 40% (95% CI: 29–51%) with a median DOR of 11.1 months (95% CI: 6.9-NR) [Sabari WCLC 2021]. 4% reached a complete response to treatment. The clinical benefit rate was 74% (95% CI: 63–83%). Median progression-free survival was 8.3 months (95% CI: 6.5–10.9) and median overall survival was 22.8 months (95% CI: 14.6-NR). Responses were seen in patients with a variety of different *EGFR* exon20ins, regardless of site of insertion type [62].

As a non-randomized single-arm study, CHRYSALIS leaves some clinical questions for the application of amivantamab across heterogeneous patient populations. Real-world analyses may allow for comparison of the study agent to current standard of care practices and may be able to examine if the efficacy endpoints would be clinically meaningful. In a real-world follow-up of 81 amivantamab-treated patients compared to 125 controls all with *EGFR* exon20ins, the authors examined a subset of patients from the CHRYSALIS trial in addition to patients from three United States based-databases (ConcertAI, COTA and Flatiron) Demographics were similar to those of the original trial with median age of 62 years, 60% female, 59% never-smokers. The population included 40% with baseline brain metastases and a median of two lines of prior therapy in metastatic disease. These demographics were similar to established characteristics of exon20ins NSCLC patients from the CHRYSALIS study. Authors showed ORR of 40% with amivantamab versus 16% in the control group [66]. Patients in the amivantamab arm demonstrated longer PFS of 8.3 versus 2.9 months (95% CI 0.34–0.65) and OS of 22.8 months versus 12.8 months (95% CI 0.31–0.77). These results were consistent in subanalysis comparing amivantamab to commonly used treatments in the control arm including non-platinum-based chemotherapy, immunotherapy, *EGFR* TKIs, and platinum-based chemotherapy.

Another real-world analysis of patients from the United States as well as Europe showed a similar response with ORR of 37% vs. 17% with amivantamab compared to control [67]. This trial looked at 349 patients, 61% of whom were female. Similarly, patients had a median of two prior lines of therapy and 26% had brain metastases at the time of enrollment. The median PFS and OS were also similar at 12.5 months and 22.8 months, respectively. This study found that clinicians prescribe multiple treatments including *EGFR* TKIs to NSCLC patients with *EGFR* exon20ins despite known poor response rates, demonstrating the need for additional treatment options and further collaboration with practitioners. These real-world analyses provide prognostic information useful for patients who may be excluded from clinical trials, such as those with brain metastases. Across heterogeneous patient populations from the United States and within the European Union, amivantamab showed statistically significant benefit in ORR, OS, and PFS. These findings support the generalizability of the original study results in *EGFR* exon20ins NSCLC.

Currently there are no approved treatments for patients with triple *EGFR* mutations. Although fourth generation *EGFR* TKIs are being studied in the context of triple mutations in cis, it will likely take several years before these agents are available for patient use [68]. A case report has described the use of amivantamab in this scenario. Briefly, the patient was diagnosed at age 67 with stage IV *EGFR* L858R NSCLC and was found to have *EGFR* T790M mutation upon progression on erlotinib as well as a G796S mutation upon progression on osimertinib. Given amivantamab’s efficacy across a range of *EGFR* mutations and benefit in chemotherapy-refractory *EGFR* exon 20 insertions, [69] it was trialed in her refractory L858R/T790M/G796S *EGFR* mutations [70]. The patient demonstrated lower symptom burden, mutation allele frequency, and CEA level with ongoing response at follow-up over 100 days later. This shows that amivantamab may be active against one of the most commonly acquired triple *EGFR* mutations in cis.

#### Safety, tolerability, and adverse events

From the *EGFR* exon20ins post platinum-based therapy cohort in the CHRYSALIS study, the most common adverse events include rash (86%), infusion-related reaction (65%), and paronychia (42%) [62]. Rare adverse events include stomatitis, pruritis, hypoalbuminemia, increased ALT, fatigue, and cellulitis. The side effects are associated with the unique properties of this drug as rash, paronychia, stomatitis, pruritis, and diarrhea resulting from *EGFR* inhibition while *MET* inhibition is associated with hypoalbuminemia and peripheral edema (Table 1). 16% of patients experienced grade 3 or higher treatment-related adverse events. The most common of which include rash (4%), infusion-related reaction (3%), and neutropenia (3%). Serious treatment-related adverse events included infusion-related reactions (2%), and diarrhea (2%). Interstitial lung disease occurred in 4% of patients. Significant side effects resulted in amivantamab dose-reduction in 13% and drug discontinuation in 4% of participants. Almost all (94%) of infusion-related reactions occurred during the first infusion.

**Table 1:** Amivantamab Side Effect Profile (based on Park et al., JCO 2021)

*EGFR*-Specific AEs	Rate	*MET*-Specific AEs	Rate
Rash	86%	Hypoalbuminemia	27%
Paronychia	45%	Peripheral edema	18%
Stomatitis	21%		
Pruritis	17%		
Diarrhea	12%		
		Both	
Infusion-Related Reactions		66%	

Signs of infusion-related reaction include chills, dyspnea, flushing, nausea, chest discomfort, and emesis. These reactions were mitigated in the CHRYSALIS trial by holding of infusion (56%), reinitiating at a slowed rate (53%), or aborting infusion (14%) [71]. As a result, amivantamab is typically administered as a slow infusion over two days for the first cycle. On day 1 amivantamab is administered at 25 mL/h for the first two hours then increased to 50 mL/h for the remainder of the 350 mg dose. With this regimen, the median time to infusion-related reaction onset is 45 min and the majority of reactions that occur are grade 1–2. No predisposing risk factors for which patients will develop infusion-related reactions were identified.

In further safety analysis of 302 patients with any *EGFR* mutation who received at least 1 dose of amivantamab, side effects occurred in approximately 20% of patients [65]. These side effects were consistent with those reported earlier and include infusion-related reactions, rash, paronychia, stomatitis, and edema. Of those with infusion-related reactions, 97% were grade 1 or 2.

A subcutaneous form of amivantamab is being studied in the PALOMA trial (NCT04606381). Preliminary results show subcutaneous route is well-tolerated and reduced infusion-related reactions to 18.2% [72]. This route allows for decreased administration time to less than 5 min and maintained approximately 65% of the bioavailability seen with intravenous dosing [73]. Saturation of free *EGFR* and *MET* receptors was seen after the first dose.

Both anti-*EGFR* and anti-*MET* therapies are associated with dermatologic toxicities. An analysis of cutaneous side effects from patients enrolled in the phase I CHRYSALIS trial noted acneiform rash and paronychia in 100% of patients [74]. Other adverse events include hypertrichosis in 50% of men, hirsutism in 80% of women, skin abrasians of scalp (71%), and skin fissure (57%). Amivantamab administered at the higher dose of 1400 mg was associated with both higher grade and more rapid skin toxicity. Secondary prevention of cutaneous manifestations should utilize tetracycline, moisturizers, and hygienic measures at least 14 days prior to treatment initiation. It is essential to ask patient about skin reactions to therapy as these can have a psychological impact.

In a small review focusing on dermatologic side effects, lesions associated with amivantamab use may appear more severe with unique features and distribution due to the dual *EGFR* and *MET* inhibition [75]. Patients were noted to have severe crusted plaques of the scalp which may be the result of *MET* activity which is known to impact follicle growth [76, 77]. The dermatologic effects of amivantamab are reduced 50% when patients use proactive therapy with moisturizers, sunscreen, topical corticosteroids, and oral tetracycline [78].

### EGFR exon 19 deletion and L858R mutation post osimertinib

This cohort was designed to combine lazertinib, a potent, CNS-penetrant, third-generation *EGFR* TKI with amivantamab which has the potential to target the two most common resistance mechanisms to TKIs – secondary *EGFR* mutations and *MET* amplification. Preliminary results presented at ASCO 2022 were from the 45 patients who were chemotherapy naïve but progressed on osimertinib. Analysis showed ORR 33% (95% CI 26–41) with PFS 5.1 months (95% CI 4.2–6.9) [79]. Median DOR was 9.6 months (95% CI 7.0-NR) and median OS 14.8 months (95% CI 12.1-NR). Of note, intracranial ORR was 26% (7/27). The most common treatment-related adverse events include infusion-related reactions (67%), paronchia (52%), and rash (44%). Grade 3 or higher adverse events seen include dyspnea (8%), infusion-related reaction (8%), and hypoalbuminemia (7%).

### NSCLC with MET exon 14 skipping mutation

Given amivantamab’s higher affinity for *MET* over *EGFR*, the phase I CHRYASLIS trial is also looking at amivantamab in *MET* amplification (Cohort MET-1) and *MET* exon 14 skipping mutations (Cohort *MET*-2). Preliminary data within the *MET* exon 14 skipping mutations cohort (n = 55) was presented at ASCO 2022. Results revealed ORR 33% (15/45) with a median PFS 6.7 months (95% CI 2.9–15.3) [72]. The response was most pronounced in treatment-naïve patients (ORR 57%) compared to those who were pretreated without *MET* inhibitors (47%) and with prior *MET* inhibitors (17%). Within responders, 10/15 demonstrated response greater than 6 months and median DOR was not reached. Most common toxicities include infusion-related reactions (69%), dermatitis (40%), and paronchia (38%). Grade 3 and higher AEs include dyspnea (7%), infusion-related reactions (5%), and hypoalbuminemia (4%). Treatment-related adverse events resulted in dose reduction or drug discontinuation in three patients.

## Discussion/future directions

Traditional *EGFR* targeted therapies such as gefitinib, erlotinib and osimertinib which are effective against those with *EGFR* sensitizing mutations, were not as effective against *EGFR* exon20ins-mutated NSCLC, leaving an unmet need for a significant percentage of lung adenocarcinoma patients with *EGFR* mutations. Approximately 90% of exon20ins mutations occur after the C-helix of the tyrosine kinase domain, wedging the C-helix in front of the drug binding pocket resulting in active kinase formation making it difficult for drug binding [9, 80]. This may explain resistance to first generation TKIs [81]. Second generation TKIs in exon20ins are limited by significant toxicity at plasma concentrations below the efficacy threshold required to inhibit signaling pathways [14]. The third generation *EGFR* TKI, osimertinib, was ineffective in *EGFR* exon20ins with a low overall response rate of 5% [81, 82]. Patients with newly diagnosed *EGFR* exon20ins-driven NSCLC have a median OS of 16.2 months (95% CI: 11.0-19.4) [83] compared to a median OS of 38.6 months in those with exon 19 deletions and 21 mutations based on the FLAURA study of front-line osimertinib [84].

Promising results from the phase I CHRYSALIS trial led to the FDA accelerated approval of amivantamab in those with *EGFR* exon20ins post platinum-based therapy. Amivantamab is the first FDA-approved bispecific molecule for treatment of solid malignancies. The investigators found an ORR of 40% with a median PFS of 8.3 months. These findings are clinically meaningful considering that relapsed metastatic or unresectable NSCLC has a 5-year survival rate of less than 10% [62]. While mobocertinib also received FDA accelerated approval for the same indication with potentially similar efficacy profile, [85] as the confirmatory phase 3 EXCLAIM-2 study did not meet its primary endpoint, Takeda has announced its voluntary withdrawal.

Further enhancing the efficacy with combination therapy with chemotherapy may be attractive to patients especially those with an initially high tumor burden. The ongoing phase III PAPILLON trial (NCT04538664) is studying the efficacy and safety of carboplatin-pemetrexed chemotherapy with or without amivantamab in the first-line treatment setting of metastatic NSCLC with *EGFR* exon20ins. This design is particularly favorable, as the standard of care first line treatment is platinum doublet, ensuring patients a “no-risk” approach, while the addition of amivantamab from first line may have a chance to induce even better efficacy results. The primary outcome of interest is PFS at 18 months with secondary outcomes including ORR, DOR, and tolerability (Table 2). Additional evidence is needed for patients with baseline brain metastases using combinations of amivantamab with chemotherapy, targeted agents, and radiation along with careful evaluation of the associated toxicity profiles.

**Table 2:** Ongoing Trials of Amivantamab

Clinical Trial	Study Drugs	Disease	Patients	Phase	Primary Outcome	Status
NCT04538664 PAPILLON	amivantamab + carboplatin + pemetrexed vs. carboplatin + pemetrexed	NSCLC	N = 300	3	PFS	Recruiting
NCT04487080 MARIPOSA	amivantamab + lazertinib vs. osimertinib vs. lazertinib	NSCLC	N = 1074	3	PFS	Active, not recruiting
NCT04988295 MARIPOSA-2	amivantamab + lazertinib + platinum chemotherapy	NSCLC	N = 600	3	PFS	Recruiting
NCT05388669 PALOMA-3	amivantamab + lazertinib	NSCLC	N = 640	3	Serum concentration	Recruiting
NCT04965090	amivantamab + Lazertinib	NSCLC	N = 40	2	CNS ORR	Recruiting
NCT05074940	amivantamab	Adenoid cystic carcinoma	N = 18	2	ORR	Recruiting
NCT05299125	amivantamab, lazertinib, carboplatin, pemetrexed	NSCLC	N = 49	2	PFS, OS	Not yet recruiting
NCT05117931	amivantamab	Esophagogastric cancer	N = 25	2	ORR	Recruiting
NCT04945733	amivantamab	Gastric or esophageal cancer	N-79	2	ORR	Recruiting
NCT05498428 PALOMA-2	amivantamab	Solid tumors	N = 260	2	ORR, adverse events	Not yet recruiting
NCT05488314	amivantamab + capmatinib	NSCLC	N = 147	1/2	Dose limiting toxicity, ORR	Not yet recruiting
NCT05379595	amivantamab	Colorectal cancer	N = 225	1b/2	ORR, dose limiting toxicity	Recruiting
NCT04077463 CHRYSALIS-2	lazertinib +/- amivantamab	NSCLC	N = 460	1/1b	Dose limiting toxicity, ORR	Recruiting
NCT04085315	amivantamab + osimertinib	NSCLC	N = 38	1/1b	Safety and tolerability	Recruiting
NCT05395052	FT536 + amivantamab and other monoclonal antibodies	Solid tumors	N = 322	1	Recommended dose, adverse events	Recruiting
NCT02609776 CHRYSALIS	amivantamab	NSCLC	N = 780	1	ORR, DOR, dose limiting toxicity	Recruiting
NCT04606381 PALOMA	amivantamab	Solid tumors	N = 196	1	Serum concentration, dose limiting toxicity	Recruiting

Amivantamb, given its broad-spectrum coverage against *EGFR* and the fact that it is a bispecific against *MET*, which alterations are known to be part of the mechanism of resistance against osimertinib, is also being evaluated in the first line setting for those with *EGFR* exon 19 deletions and L858R mutations as well as post progression on osimertinib.

Of 20 treatment-naïve patients with classical *EGFR* mutations treated with amivantamab plus lazertinib in the CHRYSALIS (NCT02609776) study, the ORR was 100% [86]. In the post osimertinib setting, preliminary data of CHRYSALIS-2 showed promising results with ORR 36%, clinical benefit rate of 58% including one complete response. Although the median DOR was not reached, 39% of participants had a durable response at a median follow-up of 8.3 months [79]. Sub-analysis of the heavily-pretreated population found ORR 29% with clinical benefit rate 55% and median DOR 8.6 months. Also, among eight patients with baseline brain lesions, antitumor activity was reported.

Currently, the CHRYSALIS-2 trial (NCT04077463) is studying amivantamab and lazertinib in *EGFR*-NSCLC (Table 2). While the phase III MARIPOSA study (NCT04487080) is investigating the safety and efficacy of amivantamab in combination with lazertinib versus lazertinib alone or lazertinib alone in NSCLC patients with classic *EGFR* mutations in the front line setting, MARIPOSA-2 study (NCT04988295) is evaluating three arms (lazertinib + amivantamab + carboplatin + pemetrexed, carboplatin + pemetrexed, amivantamab + carboplatin + pemetrexed) post progression on lazertinib in those with the classic *EGFR* mutations. The primary outcome of interest is PFS with secondary endpoints of ORR, OS, DOR, intracranial PFS among others.

The safety profile of amivantamab combined with lazertinib is similar to that of amivantamab monotherapy [86]. Most common adverse events include rash (78%), infusion-related reactions (61%), paronychia (42%), stomatitis (31%), and pruritis (24%). Grade 3 or higher adverse events were reported in 7% of participants. Similar rates of infusion-related reaction (65%), paronychia (49%), rash (41%), and stomatitis (39%) were seen in CHRYSALIS-2 [79]. This combination has the benefit of amivantamab’s activity against extracellular *EGFR* with lazertinib’s intracellular *EGFR* TKI efficacy. Lazertinib also crosses the blood-brain barrier, making this combination favorable for NSCLC patients with brain metastases who have limited effective treatment options. Within the CHRYSALIS cohort, only 7% of patients on combination therapy had documented central nervous system progression compared to 17% with amivantamab monotherapy [87]. Additional ongoing trials with amivantamab are listed in Table 2.

Currently there are no FDA approved targeted agents for *MET* amplified cancers. Although new therapeutics targeting *MET* include capmatinib and tepotinib for *MET* exon 14 skipping mutations have become available, additional treatment options are needed. Amivantamab has shown early promising data in *MET* exon 14 skipping mutations with ORR 33% in all patients and 57% in treatment-naïve patients [72]. The median PFS was 6.7 months and was generally well tolerated.

While amivantamab is an excellent agent towards *EGFR* exon20ins and beyond, we must be cognizant of the adverse event profile and the inconvenience to patients. To circumvent the infusion time (and potentially infusion related reactions), multiple studies are looking at utilizing the subcutaneous version of amivantamab in NSCLC and solid tumors (PALOMA: NCT04606381, PALOMA2 NCT05498428 and PALOMA3 NCT05388669). Preliminary results look promising with a remarkably lower rate of infusion-related reactions.

## Conclusion

The unique structural design of amivantamab with simultaneous binding to both *EGFR* and *MET* with addition of a low fructose backbone for enhanced ADCC provide increased selectivity and efficacy with decreased toxicity compared to other targeted therapies for *EGFR* exon20ins NSCLC [53]. Amivantamab, especially in combination with lazertinib and appears to have promising activity beyond *EGFR* exon20ins. These indications may include classic *EGFR* mutations in the front line setting and in the post osimertinib failure scenario as well as those with *MET* alterations. Additional studies are warranted to not only document and improve on the clinical efficacy of amivantamab in different settings but also to reduce the toxicities and inconvenience of therapy.

## Data Availability

Not applicable to this study.

## Abbreviations

ADCC: antibody dependent cell-mediated cytotoxicity
ALK: anaplastic lymphoma kinase
AKT: protein kinase B
BIM: BCL2-intearcting mediator of cell death
CI: confidence interval
CNS: central nervous system
DOR: duration of response
ERK: extracellular signal-related kinase
EGF: epidermal growth factor
EGFR: epidermal growth factor receptor
Exon20ins: exon 20 insertion mutations
FDA: federal drug administration
HGF: hepatocyte growth factor
MAPK: mitogen-activated protein kinase
MET: mesenchymal-epithelial transition factor
NSCLC: non-small cell lung cancer
ORR: overall response rate
OS: overall survival
PFS: progression free survival
PI3K: phosphatidylinositol 3 kinase
TKI: tyrosine kinase inhibitor
STAT: signal transducer and activator of transcription
